# Macular Ganglion Cell-Inner Plexiform Layer Thickness Prediction from Red-free Fundus Photography using Hybrid Deep Learning Model

**DOI:** 10.1038/s41598-020-60277-y

**Published:** 2020-02-24

**Authors:** Jinho Lee, Young Kook Kim, Ahnul Ha, Sukkyu Sun, Yong Woo Kim, Jin-Soo Kim, Jin Wook Jeoung, Ki Ho Park

**Affiliations:** 1 Department of Ophthalmology, Seoul National University College of Medicine, Seoul, Korea; 2 Department of Ophthalmology, Seoul National University Hospital, Seoul, Korea; 3 Interdisciplinary Program, Bioengineering Major, Graduate School, Seoul National University, Seoul, Korea; 4 Department of Ophthalmology, Hallym University Chuncheon Sacred Heart Hospital, Chuncheon, Korea

**Keywords:** Optic nerve diseases, Translational research

## Abstract

We developed a hybrid deep learning model (HDLM) algorithm that quantitatively predicts macular ganglion cell-inner plexiform layer (mGCIPL) thickness from red-free retinal nerve fiber layer photographs (RNFLPs). A total of 789 pairs of RNFLPs and spectral domain-optical coherence tomography (SD-OCT) scans for 431 eyes of 259 participants (183 eyes of 114 healthy controls, 68 eyes of 46 glaucoma suspects, and 180 eyes of 99 glaucoma patients) were enrolled. An HDLM was built by combining a pre-trained deep learning network and support vector machine. The correlation coefficient and mean absolute error (MAE) between the predicted and measured mGCIPL thicknesses were calculated. The measured (OCT-based) and predicted (HDLM-based) average mGCIPL thicknesses were 73.96 ± 8.81 µm and 73.92 ± 7.36 µm, respectively (*P* = 0.844). The predicted mGCIPL thickness showed a strong correlation and good agreement with the measured mGCIPL thickness (Correlation coefficient *r* = 0.739; *P* < 0.001; MAE = 4.76 µm). Even when the peripapillary area (diameter: 1.5 disc diameters) was masked, the correlation (*r* = 0.713; *P* < 0.001) and agreement (MAE = 4.87 µm) were not changed significantly (*P* = 0.378 and 0.724, respectively). The trained HDLM algorithm showed a great capability for mGCIPL thickness prediction from RNFLPs.

## Introduction

Glaucoma is the leading cause of visual impairment worldwide, affecting more than 70 million people^1^. Effective screening strategies are important, as most patients do not present any symptoms before the disease has reached the advanced stage^2,3^.

There has been remarkable progress in glaucoma screening thanks to the development of deep learning algorithms such as convolutional neural networks (CNNs) for visual recognition^4–6^. A number of studies have reported the diagnostic performance of the deep learning model as excellent in terms of area under receiver operating characteristic curve (AUC). Ting *et al*. reported on a deep learning system for multiethnic diabetic cohorts that achieved an AUC of 0.942^7^. Li *et al*. reported a deep learning model that had been trained on a very large-scale dataset with over 48,000 fundus photographs and that achieved an AUC of 0.986 for referable glaucomatous optic neuropathy^6^. However, the deep learning models of most of the previous studies require ground truth labeling by human graders. This labeling process is labor-intensive as well as subjective.

Spectral domain-optical coherence tomography (SD-OCT) is widely utilized for detection and quantitative assessment of glaucomatous structural loss of retinal nerve fiber layer (RNFL) and macular ganglion cell-inner plexiform layer (mGCIPL)^8–11^. It is useful not only for diagnosing glaucoma but also for monitoring glaucoma progression even before apparent visual field (VF) change^12,13^.

Recently, several studies have shown that RNFL thickness and minimum rim width relative to Bruch’s membrane opening (BMO-MRW) under SD-OCT can be successfully quantified from monoscopic optic disc photographs by deep learning models^14,15^. They showed the potential of deep learning models to provide quantitative information on the extent of neural damage from qualitative data (optic disc photographs). Although these studies are enlightening, mounting evidence from the investigation of glaucomatous damage implicates early macular involvement^10,16,17^. Analyses of the optic nerve head (ONH) and RNFL alone, therefore, potentially overlook glaucomatous macular damage.

Thus prompted, this study developed and validated a novel hybrid deep learning model (HDLM) to quantify mGCIPL thickness from red-free RNFL photographs (RNFLPs) so as to determine if HDLM-based RNFLP analysis could be an effective alternative to macular SD-OCT at clinics or glaucoma-screening centers where SD-OCTs are unavailable.

## Results

Eight-hundred and fifty-two (852) pairs of RNFLPs and SD-OCT scans from 441 eyes of 266 participants were enrolled. Among them, 63 image pairs were excluded: 42 for poor-quality RNFLP and SD-OCT; 11 for a poor-quality SD-OCT mGCIPL thickness map, and 10 for a poor-quality SD-OCT RNFL deviation map. The remaining 789 image pairs from 431 eyes of 259 subjects (183 eyes of 114 healthy controls, 68 eyes of 46 glaucoma suspects, and 180 eyes of 99 glaucoma patients) were examined in the ensuing analysis. The subjects’ demographic characteristics are summarized in Table 1. The mean age was 57.4 ± 13.4 (21–87) years; 106 of the subjects were men (40.9%) and 153 were women (59.1%). The spherical equivalent of refractive error ranged from −8.75 to 3.50D (−2.78 ± 3.41 D); AL, from 22.83 to 26.85 mm (24.6 ± 1.81 mm); treated IOP, from 8 to 20 mmHg (14.4 ± 2.9 mmHg); MD, from −15.52 to 4.28 dB (−2.6 ± 3.9 dB).

**Table 1:** Demographic and clinical characteristics of the eyes included in the study.

	Normal	Suspect	Glaucoma	P
No. of Eyes (patients)	183 (114)	68 (46)	180 (99)	N/A
No. of images	292	109	388	N/A
Age (years)	56.5 ± 13.5	57.2 ± 16.0	58.3 ± 12.3	0.464
Female (%)	104 (56.5%)	42 (62.7%)	105 (58.3%)	0.681
IOP (mmHg)	13.8 ± 2.5	13.6 ± 2.7	15.3 ± 3.2	0.176
SDOCT mGCIPL thickness (μm)	81.8 ± 5.4	75.6 ± 3.8	67.6 ± 6.6	<0.001
Temporal Raphe Sign Positivity	11 (3.8%)	75 (68.8%)	355 (91.5%)	<0.001
Average RNFL thickness (μm)	91.9 ± 9.8	86.2 ± 9.1	69.7 ± 9.5	0.001
SAP MD (dB)	−0.2 ± 2.0	−0.7 ± 2.3	−5.1 ± 6.0	<0.001
SAP PSD (dB)	2.2 ± 1.1	2.6 ± 1.7	6.7 ± 4.7	<0.001
SE (D)	−2.5 ± 3.5	−1.3 ± 2.3	−3.6 ± 3.4	0.717
Central corneal thickness (μm)	553.3 ± 33.1	535.5 ± 38.1	540.9 ± 37.3	0.002
Axial Length (mm)	24.8 ± 1.2	24.3 ± 0.7	24.9 ± 1.3	0.143

SD-OCT = spectral domain-optical coherence tomography; mGCIPL = macular ganglion cell-inner plexiform layer; dB = decibels; SE = spherical equivalent; D = diopters; SAP = standard automated perimetry; MD = mean deviation; PSD = pattern standard deviation.

**Figure 1:**
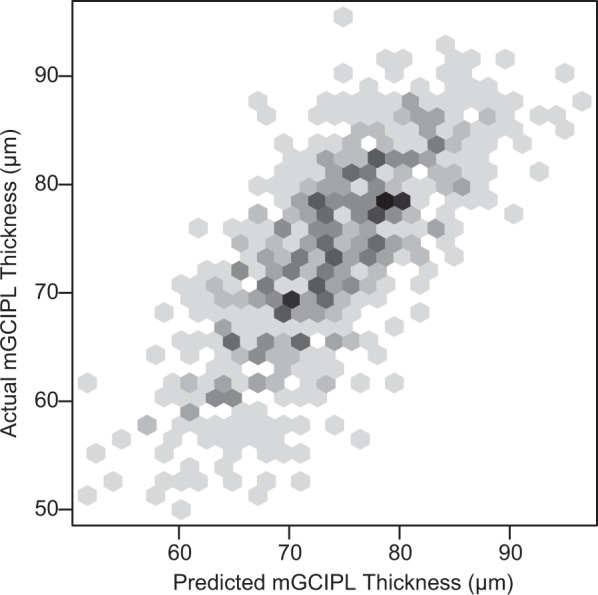
Scatterplot showing relationship between average mGCIPL thicknesses predicted by HDLM and actually measured by SD-OCT. A strong correlation was found between the predicted and the measured mGCIPL thicknesses (Pearson’s correlation coefficient = 0.739; R^2^ = 0.545). (mGCIPL = macular ganglion cell-inner plexiform layer; HDLM = hybrid deep learning model; SD-OCT = spectral domain-optical coherence tomography).

**Figure 2:**
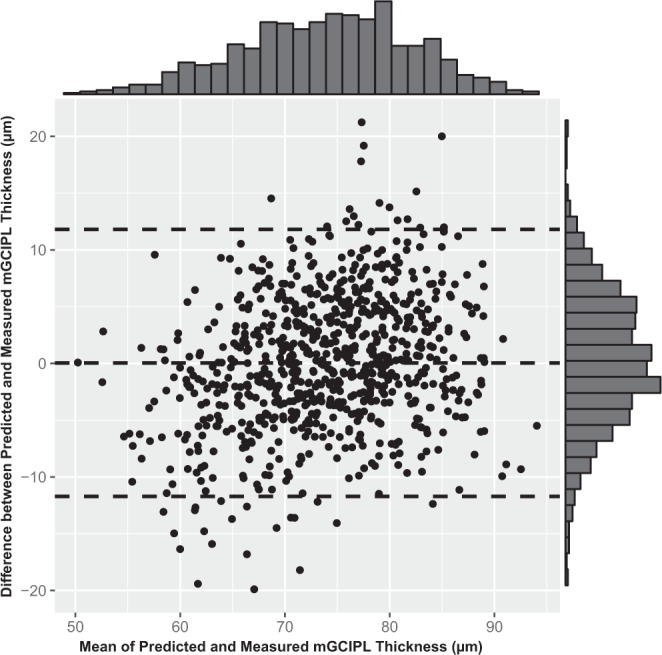
Bland-Altman plot with histograms to demonstrate agreement between prediction and measurement. The predicted mGCIPL thickness showed good agreement with the actual measurement (95% confidence limits [−11.7 µm, 11.8 µm]). (mGCIPL = macular ganglion cell-inner plexiform layer).

**Figure 3:**
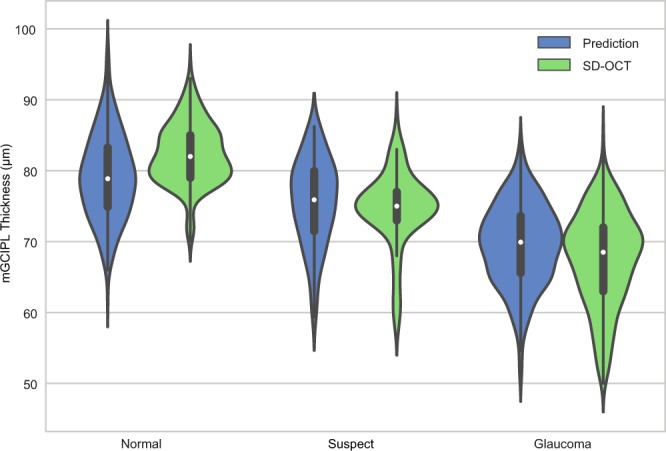
Violin plots demonstrating distribution of HDLM predictions and SD-OCT-measured average mGCIPL thicknesses in normal, suspect, and glaucomatous eyes. (HDLM = hybrid deep learning model; mGCIPL = macular ganglion cell-inner plexiform layer; SD-OCT = spectral domain-optical coherence tomography).

The measured (SD-OCT-based) average mGCIPL thickness was 73.92 ± 7.36 µm, and the predicted (HDLM-based) average mGCIPL thickness was 73.96 ± 8.81 µm (*P* = 0.844; LMM). These results indicated strong agreement (MAE = 4.76 µm). A hexbin scatterplot also demonstrated a strong correlation between the measured and predicted average mGCIPL thicknesses (Pearson’s correlation coefficient = 0.739; *R*^2^ = 0.545; Fig. 1). Figure 2 shows the Bland-Altman plot evaluation of the agreement between predictions and measurements: The 95% confidence limits of agreement ranged from −11.7 (95% CI, [−12.4, −11.0]) µm to 11.8 (95% CI, [11.1, 12.5]) µm without evidence of systemic bias (bias = 0.034 [−0.38, 0.46] µm). The distribution of the predicted and measured mGCIPL thicknesses for each group is shown as violin plots in Fig. 3. In the subgroup analysis, the measured average mGCIPL thicknesses were 81.8 ± 5.4, 75.6 ± 3.8, 67.6 ± 6.6 µm, and the predicted average mGCIPL thicknesses were 79.1 ± 6.0, 75.6 ± 6.1, 69.5 ± 5.7 µm in the normal, glaucoma suspect, and glaucoma groups, respectively.

When the ONH was masked using the Gaussian blur function, the predicted average mGCIPL thickness was 73.9 ± 5.94 µm (*P* = 0.983; LMM). The correlation (*r* = 0.713; *R*^2^ = 0.508; *P* < 0.001) was still strong and was not statistically decreased (*P* = 0.378). Also, the agreement between the measured and predicted average mGCIPL thicknesses was still good (MAE = 4.87 µm), and there was no significant difference relative to the results for RNFLP without ONH masking (*P* = 0.724).

**Figure 4:**
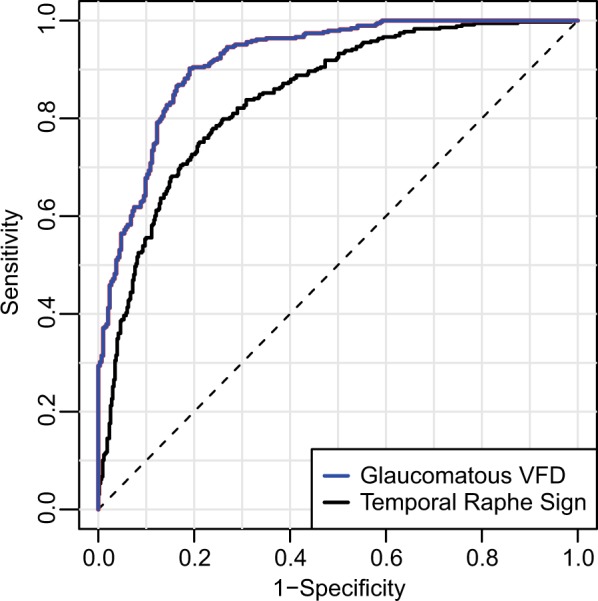
Receiver operating characteristic curve showing HDLM’s discriminating ability for glaucomatous visual field loss and temporal raphe sign. The temporal raphe sign, a step-like configuration near the temporal raphe on an HD-OCT mGCIPL thickness map, reflects the ST-IT mGCIPL asymmetry. The AUC of the HDLM’s determinations of glaucomatous VFD and temporal raphe sign positivity were 0.918 and 0.843, respectively. (HDLM = hybrid deep learning model; ST = superotemporal; IT = inferotemporal; mGCIPL = macular ganglion cell-inner plexiform layer; AUC = area under curve; VFD = visual field defect).

**Figure 5:**
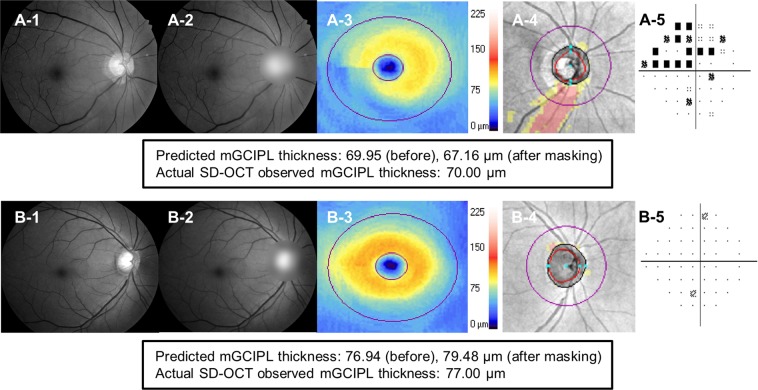
Representative glaucomatous and non-glaucomatous cases. **(A)** Glaucomatous eye of 69-year-old woman. **(A-1)** Red-free RNFLP. A distinct inferior RNFL defect is shown. **(A-2)** RNFLP with peripapillary area masking. **(A-3)** On the mGCIPL thickness map, glaucomatous inferotemporal mGCIPL thinning is evident. **(A-4)** RNFL deviation map. **(A-5)** Pattern deviation plot reveals superior hemifield defect. The mGCIPL thickness was predicted to be 69.95 μm, and the actual thickness was measured to 70.00 μm. After masking of the peripapillary area, the prediction was changed to 67.16 μm. **(B)** Healthy eye of 53-year-old man. **(B-1)** The red-free RNFLP showed no definite structural damage. The mGCIPL thickness map **(B-3)** and RNFL deviation map **(B-4)** also showed normal findings. **(B-5)** The pattern deviation plot also indicated no evidence of glaucomatous damage. The mGCIPL thickness was predicted to be 76.94 μm and 79.26 μm before and after peripapillary area masking **(B-2)**, respectively, while the actual thickness was measured to 77.00 μm. (RNFL = retinal nerve fiber layer; RNFLP = retinal nerve fiber layer photograph; mGCIPL = macular ganglion cell-inner plexiform layer; SD-OCT = spectral domain-optical coherence tomography).

**Figure 6:**
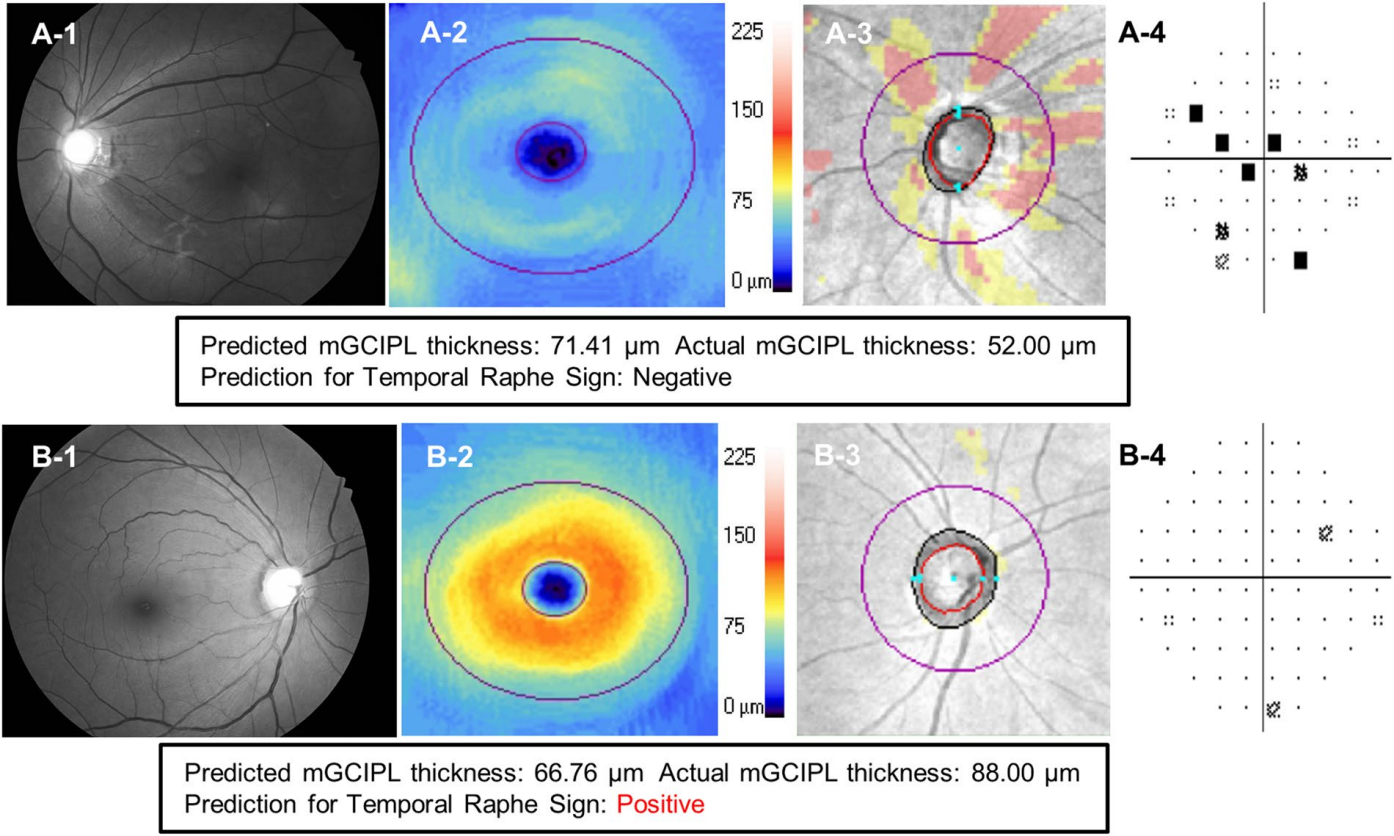
Examples of red-free fundus photographs from which mGCIPL thickness was incorrectly predicted by HDLM. (**A**) Myopic, glaucoma-suspect eye of 39-year-old woman. She had a spherical equivalent refraction of −10.1 diopters. On the red-free RNFLP **(A-1)** and RNFL deviation map in SD-OCT **(A-3)**, superior and inferior RNFL defect is observed. **(A-2)** Diffuse mGCIPL thinning is also shown on the mGCIPL thickness map. The mGCIPL thickness was predicted to be 71.41 μm, while the actual thickness measured by SD-OCT was 52.00 μm. However, the temporal raphe sign was correctly predicted to be negative. (**B**) Healthy eye of 44-year-old woman. There was no structural glaucomatous change on the RNFLP **(B-1)** or mGCIPL thickness map **(B-2)** or RNFL deviation map **(B-3)**. The visual field test also was normal **(B-4)**. The mGCIPL thickness was predicted to be 66.76 μm; however, the SD-OCT measured the mGCIPL thickness to 88.00 μm. Also, the temporal raphe sign was incorrectly predicted to be positive. (HDLM = hybrid deep learning model; RNFL = retinal nerve fiber layer; RNFLP = retinal nerve fiber layer photograph; mGCIPL = macular ganglion cell-inner plexiform layer; SD-OCT = spectral domain-optical coherence tomography).

The HDLM’s ability to differentiate eyes with glaucomatous VF loss from healthy eyes was excellent (AUC = 0.918, 95% CI [0.898–0.939]; sensitivity 0.901, 95% CI [0.852–0.945]; specificity 0.805, 95% CI [0.764–0.843]). The classifier’s classification accuracy for the three groups (normal, glaucoma suspect, glaucoma) was 81.7%. Likewise, its ability to determine temporal raphe sign positivity was good as well (AUC 0.843, 95% CI [0.816–0.870]; sensitivity 0.779 [0.737–0.821]; specificity 0.761 [0.719–0.801]). Figure 4 plots ROC curves showing the HDLM’s diagnostic ability for glaucomatous VF loss and temporal raphe sign positivity. Representative cases of correct and incorrect prediction of mGCIPL thickness by the HDLM are shown in Figs. 5 and 6, respectively.

To estimate the possibility of learning random thickness rather than true features related to mGCIPL, the efficacy of the model was further investigated with various corruption ratios. As shown in Supplementary Table S1, the R^2^ score and MAE of 5-fold CV became worse as the corruption ratio increased. With the corruption ratio ≥ 0.4, the R^2^ scores were even negative values. When the prediction error of the model is worse than just predicting the mean value, the R^2^ score can be negative^18^. Supplementary Fig. S1 depicts the relationship between the predicted mGCIPL thickness and the target thickness with the corruption ratios of 0.4 (Fig. S1A) and 0.8 (Fig. S1B). With the corruption ratio of 0.4, a weak positive correlation could still be observed, whereas no correlation was found with the corruption ratio of 0.8.

## Discussion

A novel deep learning model for quantification of mGCIPL thickness from RNFLPs was validated in this study. The predicted mGCIPL thickness using the HDLM algorithm showed strong correlation and good agreement, even after ONH shielding, with the measured mGCIPL thickness.

SD-OCT has become an essential tool for objective monitoring of glaucoma. There are several studies that demonstrated the potential of deep learning models for glaucoma diagnosis using SD-OCT data^19,20^. Going one step further, research has shown the possibility of deep learning-based quantitative prediction (RNFL thickness or BMO-MRW) beyond the limits of simple classification of disease status^14,15^. Thus prompted, we undertook a new challenge, which was to use the HDLM algorithm to convert qualitative data (RNFLP) to quantitative data (mGCIPL thickness). On RNFLPs, the mGCIPL is not noticeable by graders. Nevertheless, the HDLM predicted the mGCIPL thickness from RNFLPs with considerable accuracy.

A number of studies have found that early structural damage can occur in the macula, which fact emphasizes the necessity of macular SD-OCT for early-glaucoma detection^8–10,16,17,21^. Also, mGCIPL thickness measurement is known to have less test-retest variability than RNFL^22,23^, with comparable diagnostic performance to RNFL thickness^10,16^. Furthermore, in a previous investigation, 94.0% (78/83) of early glaucoma showed temporal raphe sign positivity^24^. Taking this into account, the HDLM, which quantitatively predicts mGCIPL thickness and precisely judges the presence or absence of the temporal raphe sign, might be a useful means of detecting early-glaucomatous change occurring prior to evident RNFL loss.

Although SD-OCT is common tool for glaucoma monitoring, fundus photography is still the main modality for glaucoma screening considering the relatively higher cost of SD-OCT. According to the results of the present study, the HDLM algorithms showed the potential to be useful tools for early-glaucoma detection in clinics or glaucoma-screening centers where SD-OCTs are unavailable. Furthermore, since this is a regression model the follows a quantitative approach, it could be expanded to detect not only glaucomatous change but also disease progression. Further study with larger datasets including longitudinal observations would help to explicate this issue.

The strong correlation between average and sectoral mGCIPL and RNFL thicknesses is already well established^25^. Hence, mGCIPL thickness prediction could be made based on the peripapillary area rather than on the macula. We used Gaussian blur to blur out the peripapillary area and performed the same training and testing process, and obtained similar results (*r* = 0.739 and 0.713, respectively). Thereby, we could deduce that the model considered the macular area as more important than the peripapillary area for prediction of mGCIPL thickness.

A brilliant study demonstrated that the deep learning model accurately predicted gender, based on only fundus photography, to an AUC of 0.97^26^. On the heat maps, the fovea turned out to be the most important area for classification of gender. We could speculate that there was some unknown clinically useful information on the macular area discovered by artificial intelligence, although we cannot fully understand the relationship of the fovea to gender currently. Similarly to that previous report, in our present study, the HDLM made good predictions of mGCIPL thicknesses while human graders struggled. The performance, furthermore, did not significantly diminish after masking of the peripapillary area, which suggests that there are currently unknown mGCIPL-associated features in the macular area of RNFLPs. Further studies investigating such important macular features certainly would be interesting.

Although we masked the peripapillary area to exclude the influence of the optic disc and circumpapillary RNFL for mGCIPL thickness prediction, it was unclear which features of RNFLPs were learned by the model to quantify mGCIPL thickness. Thus, it is not guaranteed that the model is really capturing true characteristics of the mGCIPL. The model could have learned and memorized the given thickness data. According to the work of Zhang *et al*.^27^, deep learning models that have 2 layers with a rectified linear unit as an activation function are sufficiently large to memorize (overfit) the training dataset. However, the CNN model in this study was trained only with ImageNet data with its weights frozen so as not to change with the SD-OCT thicknesses. Hence, we could assume that overfitting would not occur by the CNN part of the hybrid model. However, the subsequent SVM is also prone to possible overfitting and memorization of just random noise. We re-trained and tested the models according to the same hyperparameters with perturbing of the target thicknesses. If the model easily fits corrupted thickness data, it might just learn randomly assigned values rather than truly meaningful features. However, the performance (R^2^ score and MAE) became significantly worse with higher corruption ratios. Further research could elucidate this issue more clearly.

Our study has several limitations. First, the dataset was relatively small for training of a deep learning network, which forced us to freeze the deep learning network and to train the SVM regressor only. The accuracy of the HDLM may improve further by training of a deep learning network on a larger dataset. Moreover, most of the subjects were of Korean descent, and performance with different ethnic groups could differ. Second, we used SVM in addition to the deep learning network to achieve better performance; however, it was technically difficult to draw the heat map from it. Instead, we used Gaussian blur to mask the peripapillary area to infer the most important area for mGCIPL prediction, and obtained similar results. Third, there is a possibility that the accuracy of the model depends on the fact that the mGCIPL thickness in the present study was highly structured without any large variations. The mGCIPL thicknesses in this dataset were normally ranged in this study, which could have led to overestimation of performance. However, considering that normalization is commonly adopted in many machine-learning models to convert the distribution of an input dataset to a Gaussian distribution prior to the training process, the range and distribution of our dataset might not have severely jeopardized the generalizability. Fourth, the hybrid model may simply have learned the distribution of thicknesses rather than the actual individual thicknesses of each subject. In the violin plots in Fig. 3, the shape difference between the prediction and actual data is apparent for each group. Training and testing the model in each subgroup with a larger dataset could address this issue. Fifth, we validated only the average mGCIPL thickness prediction of the HDLM, not sector-by-sector. Minimum mGCIPL thickness is known to have the best diagnostic ability, followed by the IT sectors^16^. Future study investigating the accuracy of predicting sectoral mGCIPL thickness is warranted. Fourth, although mGCIPL thickness measurement is known to show great reproducibility comparable to RNFL^22,23^, its variability still can jeopardize the performance of the algorithm.

In conclusion, we trained the HDLM to predict the SD-OCT-measured mGCIPL thickness from RNFLPs. Despite the small size of the dataset, the model predicted mGCIPL thickness with a strong measurement correlation and agreement. Also, the HDLM showed an excellent AUC for discrimination of glaucomatous VF loss. The models also showed good ability to detect the temporal raphe sign and to differentiate normal, glaucoma suspect, and manifest glaucoma eyes. These results signify that HDLM-based RNFLP analysis has the potential to be an alternative to macular OCT at clinics or glaucoma-screening centers where SD-OCTs are unavailable.

## Methods

The present research followed the tenets of the Declaration of Helsinki, the study protocol having been approved by the local ethical committee of Seoul National University Hospital. The need for informed consent was waived as part of the study approval by the ethical committee due to the retrospective nature of the study and the fully anonymized usage of the database.

### Subjects

The subjects in the study were chosen from Seoul National University Hospital (Korea)’s Glaucoma Clinic database representative of the years 2012 to 2018. All of the subjects underwent complete ophthalmologic examinations, including best-corrected visual acuity measurement, intraocular pressure (IOP) measurement by Goldmann applanation tonometry, refractive error measurement with an autorefractor (KR-890; Topcon Corporation, Tokyo, Japan), corneal pachymetry (Pocket II Pachymeter Echo Graph; Quantel Medical, Clermont-Ferrand, France), slit-lamp biomicroscopy, gonioscopy, and dilated fundus examination as well as stereo optic disc photography. The subjects additionally underwent axial length (AL) measurement (Axis II PR; Quantel Medical, Inc., Bozeman, MT, USA) and standard automated perimetry (SAP) using the Swedish interactive threshold algorithm according to the 30-2 standard program (Humphrey Field Analyzer II; Carl Zeiss Meditec, Dublin, CA, USA).

Diagnosis of glaucoma was made based on both the structural change (e.g. glaucomatous optic disc cupping or RNFL defect) and the presence of glaucomatous VF loss on SAP, which was defined as the consistent presence of a cluster of 3 or more non-edge points on a pattern deviation plot with P < 5%, including one or more with P < 1%; a pattern standard deviation (PSD) < 5% or glaucoma hemifield test results outside the normal limits, and on the presence of glaucomatous optic disc cupping (i.e. neuroretinal rim thinning, notching, excavation) or RNFL defect. VF defects had to be repeatable on at least 2 consecutive reliable tests (false positive/negatives <15%, fixation losses <15%). Glaucoma suspects were defined as glaucomatous optic disc cupping or RNFL defect without VF defect. The appearance of the optic disc on the optic disc photography and the RNFL on the red-free RNFLP were evaluated by two glaucoma specialists (J.L., Y.K.K.) who were masked to all other information on the eyes. If the opinions on the diagnosis of glaucoma differed, the third investigator (K.H.P.) was consulted who served as an adjudicator.

The control subjects had an IOP ≤ 21 mmHg with no history of increased IOP, absence of glaucomatous disc appearance or RNFL defect, and a normal VF on SAP.

Individuals identified for exclusion showed: (1) a secondary cause of glaucomatous optic neuropathy, (2) ocular or systemic disease that may cause VF loss or other optic disc abnormalities, (3) high myopia (AL ≥ 27.00 mm) which could be accompanied by myopic maculopathy, and (4) severe media opacity that would significantly obscure optic disc photography or RNFLPs.

### RNFLP examination

Red-free RNFLPs were taken using a digital fundus camera (VX-10a; Kowa Optimed Inc., Tokyo, Japan) using a monochromatic green filter after maximum pupil dilation with tropicamide 0.5% and/or phenylephrine 2.5%. All of the subjects underwent RNFL imaging by a single operator in a dark room without any illuminating device (except for the monitor). Images carefully focused on the posterior pole of the retina at 45° field of view using the built-in split-line focusing device were obtained for the RNFLPs and reviewed on an LCD monitor^28^. Image quality of the RNFLPs were assessed based on field definition. Adequate field definition was confirmed only when (1) the entire optic disc, macula, and temporal major vascular arcades were visible in the image; (2) and there was no retinal shadow caused by small-pupil artifact^29^.

### Cirrus HD-OCT measurement

Optic-disc (optic disc cube 200 × 200 protocol) and macular scans (macular cube, 512 × 128 protocol) using high definition-OCT (HD-OCT) software (Cirrus, version 6.0; Carl Zeiss Meditec) for RNFL and mGCIPL thickness measurements, respectively, were performed. Subjects with high-quality SD-OCT images of signal strength ≥ 8 and without motion artifacts, involuntary saccade, overt poor centration, or algorithm segmentation failure were included in the study.

### Design of HDLM

In this study, a pretrained CNN for feature extraction and a support vector machine (SVM) model as the regression model were used. Since the study population was relatively small for training of the deep learning network, the weights of the pretrained CNN were frozen so as not to be trained further by our dataset. In particular, we adopted the Inception-Resnet-v2 pretrained with the ImageNet database to extract rich features from each RNFLP. This CNN has been shown to have superior Top-1 and Top-5 accuracies for object-recognition tasks compared with other CNNs such as Inception-v3^30^. Before inputting the images into the deep learning architecture, the image pixel values were scaled to within the 0–1 range and then downsized to a resolution of 224 × 224. Data augmentation (horizontal flip, horizontal shifting (<10% of image size), image scaling (95–100% of original size) was performed to make the HDLM more robust to heterogeneity. Since Inception-Resnet-v2 was originally designed for discrimination of ordinary objects rather than fundus images, we used only the convolution layers for feature extraction and discarded the final classification layer.

The feature vector extracted from the CNN was inputted to train the SVM regression model. SVM is a classical machine learning model widely used before the deep learning era. It strives to find a hyperplane that maximizes the boundaries between various types of points of data in multidimensional space using the kernel function^31^. It can be used to either regression or classification problem. A hybrid CNN-SVM classifier was previously reported to successfully recognize handwritten digits^32^. For the hyperparameter optimization, random search was performed with 5-fold cross-validation (CV). No other clinical information (e.g. age or sex) were provided to the model for training or testing.

### HDLM for discrimination of glaucomatous visual field loss

Three variant HDLMs for evaluation of glaucoma-diagnostic ability were constructed. First, we formulated a hybrid CNN-SVM classifier to discriminate eyes with glaucomatous VF loss from healthy eyes. In this model, only normal and glaucoma groups were included, the glaucoma suspects having been excluded from the training and validating process. Second, we made a similar hybrid classifier to classify all three groups including glaucoma suspect. In the second model, the number of target classes was greater than two, and thus, calculating the AUC with ROC analysis, sensitivity or specificity was unavailable. Instead, we calculated only diagnostic accuracy by 5-fold cross-validation.

### HDLM for discrimination of temporal raphe sign

Our group had already suggested the usefulness of the “temporal raphe sign”^33–35^, a step-like configuration near the temporal raphe (on the Cirrus HD-OCT mGCIPL thickness map) accounting for the well-known asymmetrical pattern of glaucomatous structural damage around the macula^36–38^. It has been demonstrated to be useful for glaucoma diagnosis, even in high-myopia patients^21,34^. Hence, as a third, variant HDLM, we also constructed an HDLM classifier to discriminate eyes with from those without the temporal raphe sign and validated its diagnostic accuracy. All the patients of the 3 groups (including glaucoma suspects) were included from the training and validating process for this model.

### Overcoming the black box problem: ONH masking

**Figure 7:**
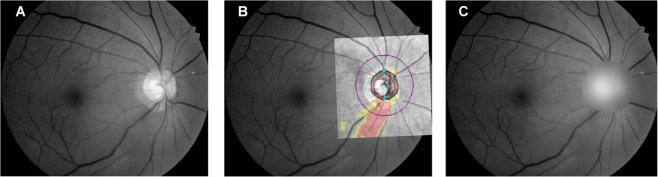
Example of peripapillary area masking on red-free RNFL photograph (RNFLP) (identical case to Fig. 5A). **(A)** Digital RNFLP. **(B)** RNFL deviation map/RNFLP overlay image with black line delineating optic disc boundary as defined by SD-OCT. **(C)** After best circle fitting by ImageJ software (National Institutes of Health), the circular area (diameter: 1.5 DD) was masked using the Gaussian blur function. (RNFL = retinal nerve fiber layer; SD-OCT = spectral domain-optical coherence tomography; DD = disc diameter).

Class activation maps (a.k.a. heat maps) of deep learning networks are drawn to visualize an area based on which a model has judged a given set of fundus photographs^14,39^. SVM models, however, are unable to generate such maps. Thus, the peripapillary area was masked on the RNFLPs and the same analyses were repeated in order to evaluate the effect of the ONH portion on mGCIPL thickness prediction with the RNFLPs. In brief, the RNFLPs were overlaid with SD-OCT RNFL deviation maps in Illustrator CS6 software (Adobe, San Jose, CA, USA) based on vascular landmarks in order to detect the objective optic disc boundary (Fig. 7)^40^. After that, ImageJ software (version 1.45 s; National Institutes of Health, Bethesda, MD, USA), was used to fit the best circle and to calculate the disc diameter (DD). Finally, the circular area including the ONH (diameter: 1.5 DD) was masked using the Gaussian blur function (kernel size: (9, 9), standard deviation: 10).

### Statistical analysis

Among the healthy, suspect, and glaucoma groups, demographic, OCT and SAP parameters were compared by the linear mixed model (LMM) accounting for multiple measurements per patient. We evaluated the performance of the HDLM for quantifying mGCIPL thickness by comparing, before and after the peripapillary area masking process, the predictions with the actual SD-OCT-measured mGCIPL thicknesses. Five-fold CV was performed to calculate the predictions from the HDLM. In the 5-fold CV, all of the feature vectors were divided into five equally sized subsets, and four of the five arms were used to train the SVM model followed by calculation of the diagnostic performance using the remaining arm. The process was repeated five times to assure that each arm was used as a validation set once. On the 5-fold CV, patient-wise split was used to prevent RNFLPs of the same patient from being split into both the training and testing arms. The mean absolute error (MAE) of the predictions as well as the Pearson’s correlation coefficients were calculated and compared before and after masking of the peripapillary area.

The discriminating ability for glaucomatous and healthy eyes was determined by the area under curve (AUC) of the receiver operating characteristic (ROC) analysis with 95% confidence intervals (CI). The sensitivity and specificity values were computed at the optimal cut-off value that maximized the Youden index (obtained as *J* = [sensitivity + specificity − 1]). To account for the use of multiple images of both eyes of the same participant in the analyses, a bootstrap resampling procedure was used to compute P values and confidence intervals, where the cluster of data for each participant was considered as the unit of resampling to adjust for standard errors^41^.

In addition, deep learning models are known to easily fit to given training data, which can lead to generalization error. It is known that many deep learning models (even consisting of only 2 hidden layers) can generate any given functions including completely unstructured random noise^27^. To confirm that the model learned truly clinically meaningful features from RNFLPs, we intentionally perturbed the target SD-OCT thicknesses with normally distributed noise (mean 30.0, standard deviation 10.0)^27^. Target thickness was calculated by weighted average of the actual SD-OCT thickness and the noise value with corruption ratio (e.g. if the corruption ratio was 0.2, the target mGCIPL thickness was computed by [actual thickness ∙ 0.8 + noise value ∙ 0.2]). We inspected the R^2^ score and MAE with the corruption ratio, varying the level of corruption from 0 (no corruption, actual thickness) to 1 (complete noise).

All of the statistical analyses were performed using R software (version 3.5.2) for statistics. P values less than 0.05 were considered statistically significant. The data ranges were recorded as mean ± standard deviations.

### Meeting presentation

This work was presented as e-Poster at the American Academy of Ophthalmology Annual Meeting 2019 in San Francisco.

## Supplementary information

Supplementary information

## Data Availability

The dataset generated during the current study is available from the corresponding author on reasonable request.
