# Supplementary material for: Macular Ganglion Cell-Inner Plexiform Layer Thickness Prediction from Red-free Fundus Photography using Hybrid Deep Learning Model
Source: Sci Rep. 2020 Feb 24;10:3280. doi: 10.1038/s41598-020-60277-y (PMC7039950; doi:10.1038/s41598-020-60277-y)

Macular Ganglion Cell-Inner Plexiform Layer Thickness Prediction from Red-free Fundus Photography using Hybrid Deep Learning Model

Jinho Lee[1,2], Young Kook Kim[1,2], Ahnul Ha[1,2], Sukkyu Sun[3], Yong Woo Kim[1,2], Jin-Soo Kim[4], Jin Wook Jeoung[1,2], and Ki Ho Park[1,2]

Supplementary Table S1. R^2 score and mean absolute error (MAE) according to various corruption ratios

**Corruption ratio**	**0.2**	**0.4**	**0.6**	**0.8**	**1.0**
R^2 score	0.460	0.172	-0.163	-0.302	-0.291
Mean absolute error (μm)	4.84	4.97	5.90	7.31	8.83

Supplementary Figure S1. Scatterplots showing relationship between predicted mGCIPL thickness and target thickness in regard to corruption ratio. The target thickness was calculated by the weighted average of the actual SD-OCT thickness and the noise value with the corruption ratio. **(A)** With the corruption ratio of 0.4, there was still a weak correlation between the prediction and target thicknesses. The R^2 score was 0.172 and the mean absolute error (MAE) was 4.97 µm. **(B)** When the corruption ratio was increased to 0.8, no correlation was observed (R^2 score: -0.302, MAE: 7.31 µm).

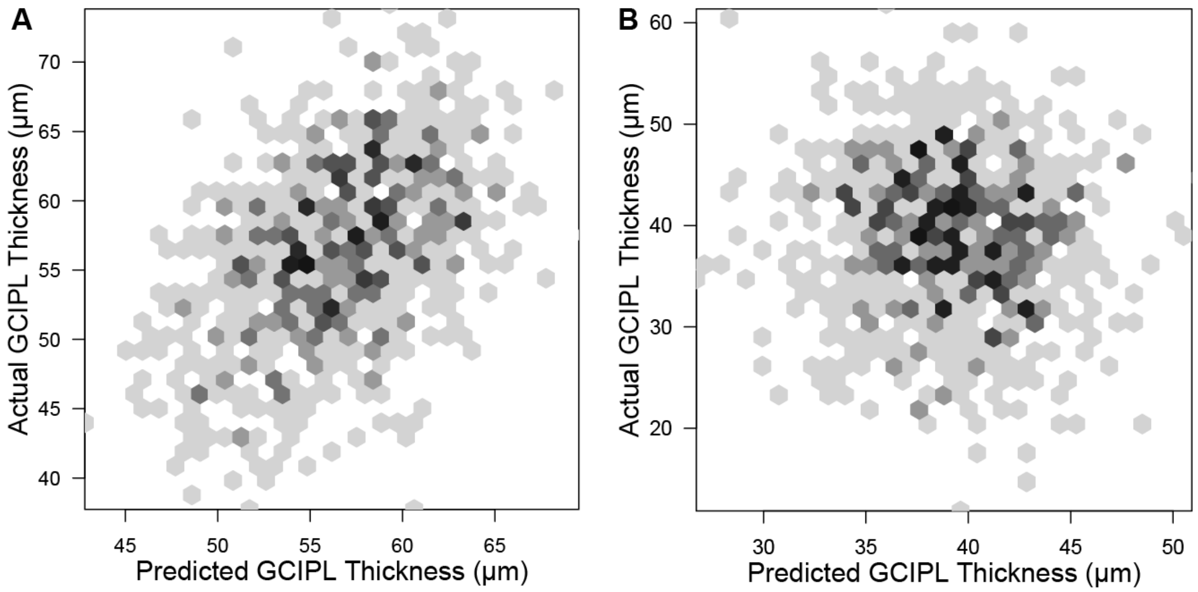

Supplement: Supplementary file 1 — Supplementary information [file 41598_2020_60277_MOESM1_ESM.pdf]
